# Resolvin D1 reverses chronic pancreatitis-induced mechanical allodynia, phosphorylation of NMDA receptors, and cytokines expression in the thoracic spinal dorsal horn

**DOI:** 10.1186/1471-230X-12-148

**Published:** 2012-10-23

**Authors:** Feng Quan-Xin, Feng Fan, Feng Xiang-Ying, Li Shu-Jun, Wang Shi-Qi, Liu Zhao-Xu, Zhang Xu-Jie, Zhao Qing-Chuan, Wang Wei

**Affiliations:** 1 Xijing Hospital of Digestive Diseases, the Fourth Military Medical University, No. 127 West Changle Road, Xi’an, 710032, China; 2 Department of Anesthesiology, School of Stomatology, the Fourth Military Medical University, No. 145 West Changle Road, Xi’an, 710032, China

**Keywords:** Chronic pancreatitis, Pain, Spinal dorsal horn, Resolvin D1, NMDA Receptor

## Abstract

**Background:**

We previously reported that immune activation in the spinal dorsal horn contributes to pain induced by chronic pancreatitis (CP). Targeting immune response in the CNS may provide effective treatments for CP-induced pain. Recent findings demonstrate that resolvin D1 (RvD1) can potently dampen inflammatory pain. We hypothesized that intrathecal injection of RvD1 may inhibit pain of CP.

**Methods:**

Rat CP model was built through intrapancreatic infusion of trinitrobenzene sulfonic acid (TNBS). All the rats were divided into three groups: TNBS, sham, and naïve controls and were further divided for intrathecal RvD1 administration. Pain behavior of rats was tested with von Frey filaments. Anxiety-like behavior and free locomotor and exploration of rats were evaluated by open field test and elevated plus maze. Pancreatic histology was evaluated with hematoxylin and eosin staining. Phosphorylation of NMDA receptor and expression of inflammatory cytokines were examined with Western blot, real-time RT-PCR and ELISA.

**Results:**

Behavioral study indicated that compared to the vehicle control, RvD1 (100 ng/kg) significantly decreased TNBS-induced mechanical allodynia at 2 h after administration (response frequencies: 49.2 ± 3.7% *vs* 71.3 ± 6.1%), and this effect was dose-dependent. Neither CP nor RvD1 treatment could affect anxiety-like behavior. CP or RvD1 treatment could not affect free locomotor and exploration of rats. Western blot analysis showed that compared with that of naïve group, phosphorylated NR1 (pNR1) and pNR2B in TNBS rats were significantly increased in the spinal cord (pNR1: 3.87±0.31 folds of naïve control, pNR2B: 4.17 ± 0.24 folds of naïve control). Compared to vehicle control, 10 ng/kg of RvD1 could significantly block expressions of pNR1 (2.21 ± 0.26 folds of naïve) and pNR2B (3.31 ± 0.34 folds of naïve). Real-time RT-PCR and ELISA data showed that RvD1 (10 ng/kg) but not vehicle could significantly block expressions of TNF-alpha, IL-1beta and IL-6. In addition, RvD1 did not influence pain behavior, NMDA receptor phosphorylation or cytokines production in sham-operated rats.

**Conclusions:**

These data highly suggest that RvD1 could be a novel and effective treatment for CP-induced chronic pain.

## Background

Pain is the primary complain of patients with visceral organ disorders. Recurrent and serious abdominal pain is one of the most common symptoms in chronic pancreatitis (CP) [1]. Lots of studies have been performed to explore the underlying mechanisms of CP-induced pain, while most of the work is focused on pathological changes in the pancreas tissue [2]. However, it is reported that pain processing in the central nervous system (CNS) is also abnormal in CP-related neuropathic pain disorders [3]. It is also suggested that pancreatic neuropathy could bring “neural remodeling” [4]. These results highly suggest that neuroplastic changes in the CNS are probably important contributors to the CP-induced chronic pain. We previously showed that astrocytes were activated and inflammatory cytokines were up-regulated in the spinal cord in a rat CP model induced by intrapancreatic infusion of trinitrobenzene sulfonic acid (TNBS) [5]. These data suggest that neuron-immune interactions in the spinal dorsal horn play a pivotal role in neuroplastic changes and CP-induced pain. More importantly, targeting immune response in the CNS may provide some novel and effective treatments for CP-induced pain.

Resolvins are biosynthesized from omega-3 fatty acids docosahexaenoic acid (DHA) and eicosapentaenoic acid (EPA), and show remarkable potency in resolving inflammation-related diseases [6]. Recent studies suggested that resolvin D1 (7S,8R,17S-trihydroxy-4Z,9E,11E,13Z,15E,19Z-docosahexaenoic acid, RvD1) exhibited potent anti-inflammatory actions in rodent models of inflammation [7]. Interestingly, RvD1 also reduced pain response in animal models of inflammatory pain. Intraplantar RvD1 post-treatment reduced complete Freund’s adjuvant (CFA)-induced mechanical hyperalgesia and allodynia [8]. In addition, intrathecal post-treatment with RvD1 also rapidly reduced CFA-induced heat and mechanical hypersensitivity [9]. These data suggest that RvD1 may provide effective anti-allodynia treatment on pain. However, whether and how RvD1 works on inflammatory visceral pain, such as pain of CP, is to be determined.

In the present study, we first investigated the behavioral changes of rats with CP following intrathecal injection of RvD1. Then we detected the phosphorylation of NMDA receptors, which play essential roles in central sensitization of pain signal processing. We further explored the production of inflammatory cytokines in the spinal cord of rats with CP after RvD1 treatment.

## Results

### Intrathecal RvD1 injection attenuated CP-induced mechanical allodynia

**Figure 1:**
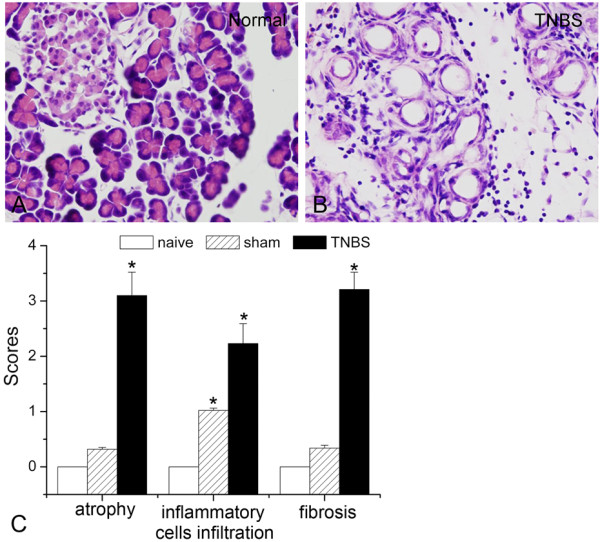
**Trinitrobenzene sulfonic acid (TNBS) infusion-induced rat chronic pancreatitis.** (**A**-**B**) HE staining of the pancreas in (**A**) normal and (**B**) TNBS infused rats. (**C**) The severity of CP was morphological assessed by semiquantitative scoring: graded glandular atrophy (0–3); intralobular, interlobular and periductal fibrosis (0–3); inflammatory cells infiltrations (0–3). BL: baseline. * *P*<0.05 compared with that of naïve group.

We previously reported that TNBS could successfully induce histological changes of pancreas at 5 w after surgery, which included acinar atrophy, inflammatory infiltration, and periductular and intralobular fibrosis, stromal proliferation (Figure 1). CP-induced persistent mechanical allodynia is characterized by increase of abdomen response frequencies (RFs) [3,10]. We previously observed that rats with CP showed persistent mechanical hypersensitivity in the abdomen [11]. The mechanical allodynia was evident 1 w after TNBS infusion and persistent up to 5 w (data not shown).

**Figure 2:**
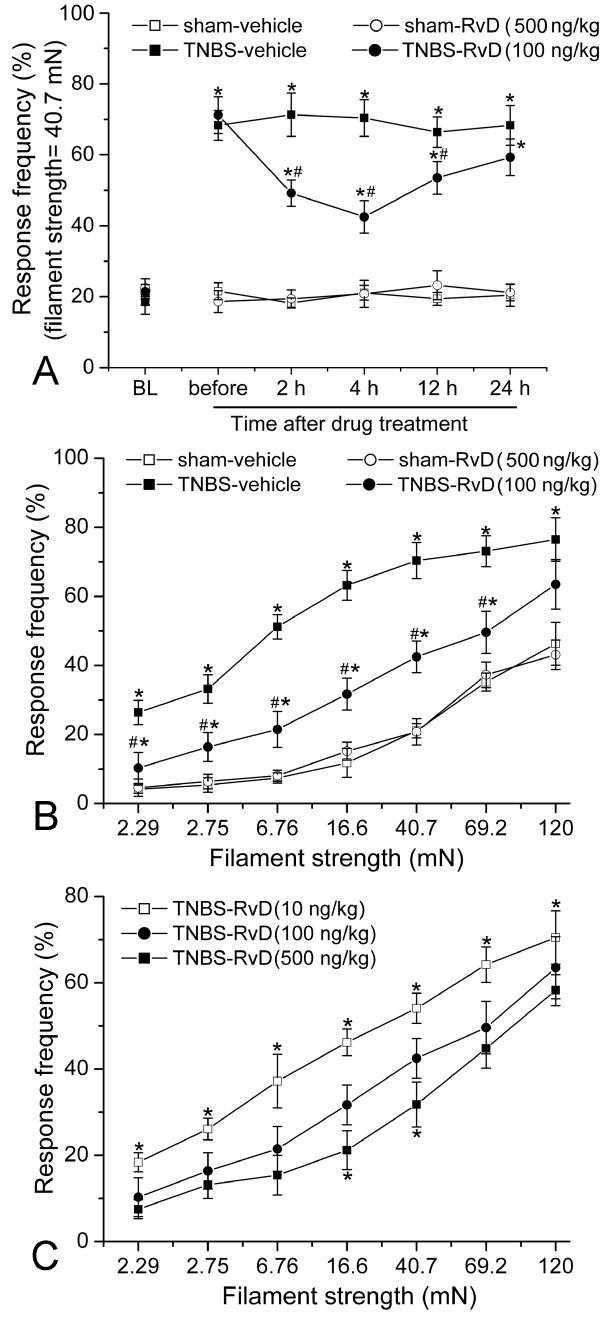
**Intrathecal administration of resolvin D 1 (RvD1) attenuates mechanical allodynia induced by CP.** (**A**) Response frequencies to mechanical stimulation of the abdomen with the 40.7 mN of von-Frey filaments at different time points after RvD1 (or vehicle) administration. BL: baseline. (**B**) Response frequencies to mechanical stimulation of the abdomen with von-Frey filaments of various strengths at 4 h after RvD1 (or vehicle) treatments. *, # *P*<0.05 compared with that of sham-vehicle or TNBS-vehicle group, respectively. (**C**) TNBS-induced allodynia was significantly attenuated by RvD1 in a dose-dependent manner. * *P*<0.05 compared to 100 ng/kg group.

To examine the anti-allodynia effect of RvD1 on CP-induced neuropathic pain, we intrathecally injected RvD1 at 5 w after TNBS infusion and observed the behavioral consequences. Compared to the vehicle control group, RvD1 (100 ng/kg) significantly decreased RFs at 2 h after administration (49.2 ± 3.7% *vs* 71.3 ± 6.1%, *P* < 0.05, Figure 2A). The anti-allodynia effect of RvD1 lasted at least for 12 h and almost disappeared at 24 h after administration. Then we tested the pain behavior with different strength of von Frey filaments (2.29, 2.75, 6.76, 16.6, 40.7, 69.2 and 120 mN) at 4 h after administration to further confirm the above data. Compared to the vehicle, RvD1 (100 ng/kg) significantly decreased RFs stimulated by most of the von Frey filaments (*P* < 0.05, Figure 2B). We further observed that TNBS-induced allodynia was remarkably attenuated by RvD1, in a dose-dependent manner (Figure 2C). However, RvD1 did not influence RFs of sham operated rats. There was no significant difference in RFs between sham-vehicle and sham-RvD1 group, at any time points after administration, or stimulated by any strength of filaments.

**Figure 3:**
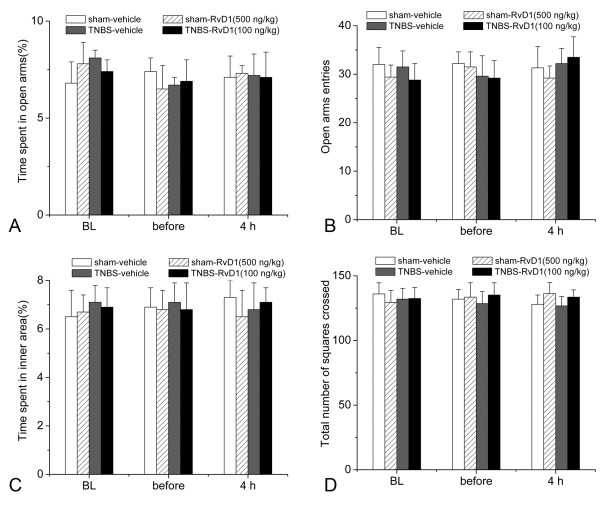
**Effects of TNBS infusion and RvD1 administration on elevated plus-maze test and open field.** Neither TNBS infusion nor RvD1 administration affected the percentage of time the test rats spent in the open arm and percentage of open arm entries in the EPM test (**A** and **B**), or percentage of time in the inner square, total number of squares in the OFT (**C** and **D**). BL: baseline.

In addition, elevated plus-maze test showed that the percentage of open-arm time and entries in rats of CP showed no significant difference compared to the sham rats. Furthermore, after treatment with RvD1, no significant difference could be detected in the percentage of open-arm time and entries between different groups (Figure 3A and B), suggesting that neither CP nor RvD1 treatment could affect anxiety-like behavior. Open field test showed no significant difference in the percentage of time in the inner squares or the total number of squares crossed could be detected among all the groups, after different treatments (Figure 3C and D). These data indicated that CP or RvD1 treatment could not affect free locomotor and exploration of rats.

### RvD1 reversed CP-induced phosphorylation of NMDA receptor NR1 and NR2B subunit in the thoracic spinal dorsal horn

**Figure 4:**
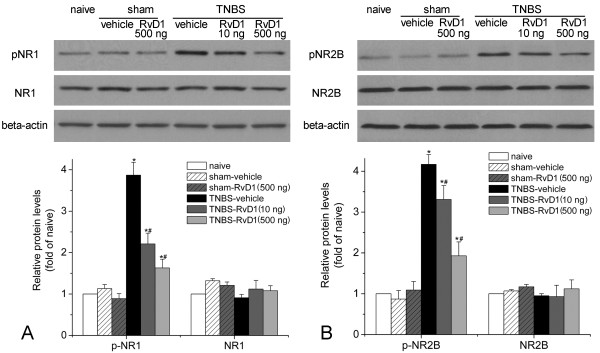
**RvD1 reverses phosphorylation of NMDA receptor subunit NR1 (A) and NR2B (B).** Data are expressed as fold changes of naïve control group. Compared with that of naïve or sham group, pNR1 and pNR2B in TNBS-vehicle were significantly increased in the spinal cord. Compared to vehicle control, RvD1 could significantly block expressions of pNR1 and pNR2B. *, # *P*<0.05 compared to sham-vehicle or TNBS-vehicle group, respectively.

**Figure 5:**
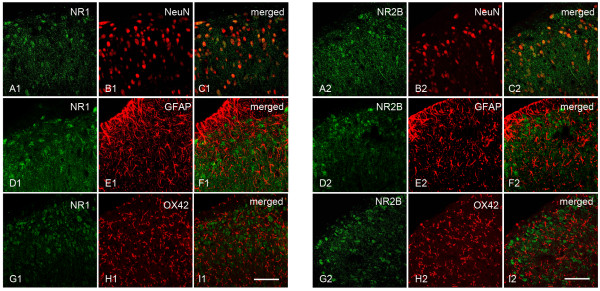
**Colocalization of NMDA receptor subunits NR1 (A1-I1) and NR2B (A2-I2) with neuronal and glial markers in dorsal horn.** Immunofluorescent colocalization of green reaction products for NR1/NR2B and red products for the neuronal marker NeuN, the astrocyitc marker GFAP, and the microglial marker OX42. Scale bars = 300 μm in I1 and I2, applied in A1-I1 and A2-I2, respectively.

Recent studies suggested that resolvins could suppress the TNF-alpha-evoked NMDA receptor hyperactivity in spinal dorsal horn neurons [9]. Western blot analysis indicated that in naïve and sham operated rats, pNR1 and pNR2B expressions in the thoracic spinal dorsal horn were very low (Figure 4). Compared with that of naïve or sham group, pNR1 and pNR2B in TNBS-vehicle were significantly increased in the spinal cord (pNR1: 3.87±0.31 folds of naïve control, pNR2B: 4.17 ± 0.24 folds of naïve control, *P*<0.05, Figure 4). Intrathecal injection of RvD1 (500 ng/kg) did not influence phosphorylation of NR1 (0.89 ± 0.12 folds of naïve) or NR2B (1.09 ± 0.21 folds of naïve) in sham operated rats. However, compared to vehicle control, 10 ng/kg of RvD1 could significantly block expressions of pNR1 (2.21 ± 0.26 folds of naïve) and pNR2B (3.31 ± 0.34 folds of naïve). In addition, 500 ng/kg of RvD1 could further decrease the phosphorylation of NR1 (1.63 ± 0.22 folds of naïve) or NR2B (1.93 ± 0.33 folds of naïve). In contrast, the expressions of total NR1 or NR2B were unchanged, either after TNBS infusion, or treated with RvD1. With double immunofluorescent histochemistry, we observed that NR1 and NR2B subunits of NMDA receptor were exclusively expressed on neurons, but not on astrocytes or microglia in the thoracic spinal dorsal horn (Figure 5). These data indicated that RvD1 could significantly modulate neuronal NMDA receptor activities and influence nociceptive transmission.

### RvD1 significantly reversed CP-induced cytokines expression

**Figure 6:**
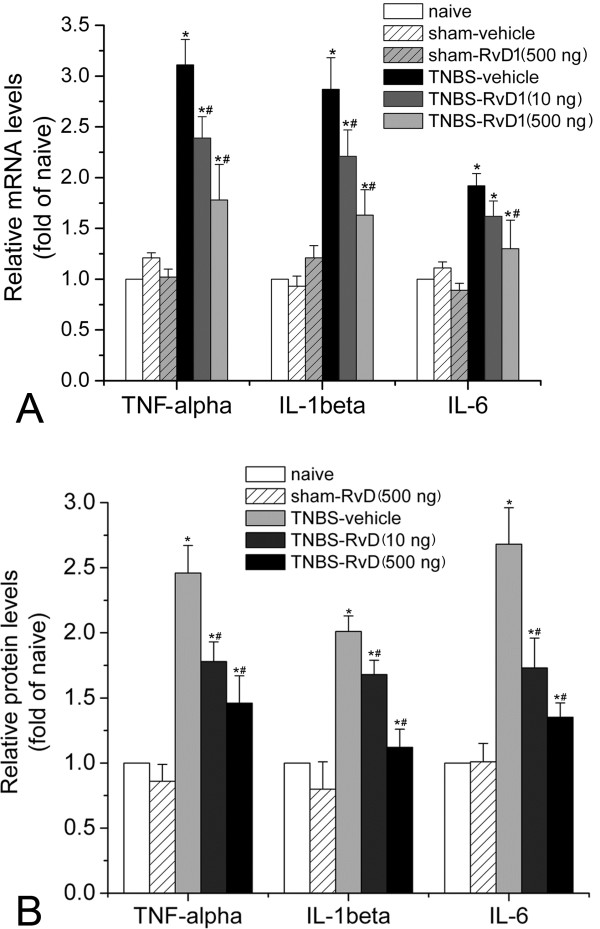
**RvD1 inhibits cytokine expressions revealed by real-time PCR (A) and ELISA (B).** Changes of TNF-alpha, IL-1beta, and IL-6 at mRNA and protein levels were determined in the T10 region of the dorsal spinal cord of CP or sham rats. Data are expressed as fold changes of naïve control group. TNBS infusion induced a remarkable increase of TNF-alpha, IL-1beta, and IL-6 compared with those of naïve groups. In addition, RvD1 could significantly decrease those expressions. *, # *P*<0.05 compared to sham-vehicle or TNBS-vehicle group, respectively.

We previously observed that various cytokines were significantly up-regulated in the spinal dorsal horn of pancreatic rats [11]. Considering the anti-inflammatory nature of RvD1, we then examined whether intrathecal injection of RvD1 could block the up-regulation of inflammatory cytokines in the spinal dorsal horn of CP rats. A significant up-regulation of cytokines was observed after CP-induced chronic pain. Real-time PCR study showed that in the TNBS-vehicle group, we observed significant increases of TNF-alpha (3.11 ± 0.25 folds of naïve, *P*<0.05), IL-1beta (2.87 ± 0.31 folds, *P*<0.05), and IL-6 (1.92 ± 0.12 folds, *P*<0.05) compared with those of naïve and sham groups. Intrathecal injection of RvD1 (10 ng/kg) but not vehicle could significantly block expressions of TNF-alpha (2.39 ± 0.21 folds of naïve, *P*<0.05), IL-1beta (2.21 ± 0.26 folds, *P*<0.05), and IL-6 (1.62 ± 0.15 folds, *P*<0.05). In addition, 500 ng/kg of RvD1 could further decrease the TNF-alpha (1.78 ± 0.35 folds of naïve, *P*<0.05), IL-1beta (1.63 ± 0.25 folds, *P*<0.05), and IL-6 (1.30 ± 0.28 folds, *P*<0.05) (Figure 6A). ELISA analysis showed similar data. TNBS infusion induced a remarkable increase of TNF-alpha (2.46 ± 0.21 folds of naïve, *P*<0.05), IL-1beta (2.01 ± 0.12 folds, *P*<0.05), and IL-6 (2.68 ± 0.28 folds, *P*<0.05) compared with those of naïve groups. In addition, RvD1 could significantly decrease those expressions (Figure 6B). These data indicated that intrathecal injection of RvD1 could significantly block TNBS-infusion induced up-regulation of inflammatory cytokines in the spinal dorsal horn.

## Discussion

RvD1 is a kind of resolvins, which were originally isolated in exudates formed in the resolution phase of acute inflammation in both rodents and humans [12]. Recent studies suggest that resolvins could be effective treatments for inflammatory and postoperative pain [8,9]. It is suggested that resolvins could reduce inflammatory pain via peripheral and central actions. Intraplantar RvD1 pretreatment reduces formalin-induced spontaneous pain and prevents carrageenan-induced hyperalgesia [8]. Intrathecal post-treatment with RvD1 could reduce CFA-induced mechanical hypersensitivity [9]. In the present study, we observed an obvious central effect of RvD1 on CP pain. Recent studies suggest that in addition to the inflammatory response in the inflamed tissue, central immune response is also critical in the process of chronic pain [13,14]. In the case of CP-induced pain, we previously observe that astrocytes are remarkably activated in the spinal dorsal horn and actively contribute to pain behavior [5]. We further find that toll-like receptor 3 is also up-regulated and mediates astrocytic activation [11]. A recent study also reports that microglia are also activated in the dorsal horn of rats with CP [15]. These findings support our notion that central inflammatory response plays pivotal roles in CP-induced pain. And that is probably how intrathecal injection of RvD1 works on the CP pain. However, the role of intra- or peri-pancreatic administration of RvD1 on CP and pain behavior is to be determined. In the present study, we administered RvD1 intrathecally at very low doses, which could not easily affect inflammation in the pancreas, especially when we injected RvD1 at 5w after induction of inflammation. That is why we believe that the analgesic effect of RvD1 is directly through spinal dorsal horn.

We then explore the underlying mechanism of the anti-allodynia effect of RvD1. Gαi-associated ChemR23 is the receptor for resolvins [16]. It is indicated that ChemR23 has a broad expression in various cell types, including spinal dorsal horn neurons [17]. Activation of NMDA receptors in the spinal neurons plays an essential role in central sensitization and thus chronic pain [18]. Functional NMDA receptors are heteromeric complexes including the NR1 subunit and one or more of the NR2A-D subunits [19]. NR2B subunit has a relatively restricted distribution in the superficial spinal dorsal horn which has more important correlations with nociceptive transmission [20]. It is reported that intrathecal injection of NMDA receptor antagonists could inhibit CP pain [21]. In the present study, we observed a significant up-regulation of phosphorylation of NMDA receptor NR1 and NR2B subunit in the thoracic spinal dorsal horn neurons. This is by far the first direct evidence of NMDA receptor phosphorylation in the pain of CP. Furthermore, we observe that RvD1 treatment could reverse CP-induced NMDA receptor phosphorylation. It is reported that resolvins could significantly block NMDA receptor phosphorylation induced by TNF-alpha [9]. Thus, it is conceivable that RvD1 may abrogate central sensitization via inhibiting phosphorylation of NMDA receptor in the spinal dorsal horn of rats with CP.

Tissue inflammation produces cytokines in spinal glial cells, which drive central sensitization by increasing excitation and decreasing inhibition in dorsal horn neurons [22]. It has been reported that spinal microglia contribute to the genesis of pain induced by CP [15]. Interestingly, the resolvins receptor ChemR23 is also expressed on microglia [17]. It is reasonable to suggest that RvD1 may also dampen pain through inhibiting glial activation and production of inflammatory cytokines. In the present study, we observed that RvD1 treatment could significantly inhibit production of spinal TNF-alpha, IL-1beta, and IL-6 induced by CP. We previously observed in a neuropathic pain model that spinal IL-1beta is mainly released by spinal astrocytes but not by microglia [23]. So probably, spinal astrocytic activation could also be hampered by RvD1 administration.

Importantly, it should be mentioned that the inhibitive effect of RvD1 on phosphorylation of NMDA receptors and on production of cytokines could be tightly related. It is reported recently that NR2B subunit phosphorylation may contribute to the release of cytokines from spinal astrocytes [23]. In addition, IL-1beta could positively modulate NMDA receptor function [24]. This positive feedback circuit enlarges the effect of primary inflammation on nociception and makes it more difficult to develop a clinical therapy for pain of CP [13]. Interestingly, RvD1 could block these two key factors and thus block the positive feedback circuit. It thus assures the preonunced anti-allodynia effect of RvD1 on CP-induced chronic pain.

## Conclusions

We demonstrate that intrathecal injection of RvD1 could significantly attenuate pain of CP, in a dose-dependent manner. We further show that intrathecal injection of RvD1 could remarkably reverse NMDA receptor phosphorylation and cytokines expression in the spinal dorsal horn of CP rats. These data highly suggest that RvD1 could be a novel and effective treatment for CP-induced chronic pain.

## Methods

### Induction of pancreatitis

Male *Sprague–Dawley* rats (250-300 g) were used in the present study. All experimental procedures received approval from the Animal Use and Care Committee for Research and Education of the Fourth Military Medical University (Xi’an, P. R. China) and also the ethical guidelines to investigate experimental pain in conscious animals.

TNBS-induced rat model of pancreatitis was built according to our previous studies [5,11]. Briefly, the common bile duct was closed temporarily near the liver with a small vascular clamp. A blunt 28 gauge needle with polyethylene (PE10) tubing attached was inserted into the duodenum. TNBS solution (0.5 ml, 2%) in 10% ethanol in phosphate buffered saline (PBS, pH 7.4) was infused into the pancreatic duct over a period of 2–5 min. The hole in the duodenum was sutured and the vascular clamp was removed restoring the bile flow. All the procedures in the sham group were same as that in the TNBS group, except that the same volume of normal saline instead of TNBS was infused into the duct.

### Administration of drugs

Laminectomy was performed at the level of the thoracic vertebrae under pentobarbital anesthesia (45 mg kg^-1^, *i.p.*). A PE10 catheter (I.D. 0.28 mm and O.D. 0.61 mm) was passed caudally from the T9 to the T12 level of the spinal cord. Two centimeters of the free ending was left exposed in the upper thoracic region. Rats were allowed to recover for a period of 3 - 5 days before further use. Only the animals judged as neurologically normal and that showed complete paralysis of the tail and bilateral hind legs after administration of 2% lidocaine (10 μl) through the intrathecal catheter were used for the following experiments. RvD1 (Cayman Chemical Co., Ann Arbor, MI, USA) was stored at -80°C. Immediately before use, RvD1 was directly diluted to working concentration with the bath solution and briefly sonicated.

All the rats were divided into three groups: TNBS (n = 42), sham (n =30), and naïve controls (n = 6). At 5 w after TNBS model, rats in TNBS (n = 36) and sham group (n = 24) were each further divided for drug injection: TNBS-RvD1 (or sham- RvD1) group: RvD1 was intrathecally injected on TNBS-infused (10 ng/kg, n= 6; 100 ng/kg, n= 12; or 500 ng/kg, n = 6; total volume = 10 μl) or sham operated rats (500 ng/kg, n =12); TNBS-vehicle (or sham-vehicle) group: 10 μl of bath solution was injected on TNBS-infused (n = 12) or sham operated rats (n = 12). After behavioral tests, all the rats including naïve, TNBS, and sham rats (n = 6 each) without intrathecal injection were sacrificed for further experiments.

### Pain behavioral test

Behavioral testing was performed according to our protocol reported previously [5]. Before test, the belly skin was shaved and area designated for stimulation was marked. Rats were placed in a plastic cage with a mesh floor and were given 30 min for adaptation. In the time course study, pain behavior was evaluated before and 2 h, 4 h, 12 h, 24 h after drug injection with a single strength of von Frey filaments (Stoelting, Kiel, WI, USA) (40.7 mN). In the force-related study, pain behavior was evaluated at 4 h after drug injection with a series of von Frey filaments (2.29, 2.75, 6.76, 16.6, 40.7, 69.2 and 120 mN). Von Frey filaments were applied from underneath through the mesh floor, in ascending order to the abdominal area at different points on the surface. A single trial consisted of 10 applications each for 1–2 s with a 15 s interval between applications allow the animal to cease any response and return to a relatively inactive position. A positive response consisted of the rat raising its belly (withdrawal response). The data were expressed as a percentage of the positive responses with each filament for each rat.

### Elevated plus-maze test

Elevated plus-maze test was performed before and 4 h after RvD1 treatment to evaluate whether CP-induced pain/RvD1 could influence anxiety-like behavior. After adaptation for 1 d, rats were placed in the middle of maze apparatus, and the behavior has been recorded by a video camera for 5 min. Data were expressed as the percentage of time spent in the open arms [100 × (time in the open arms/total time spent in both arms)] and percentage of open-arm entries as an index of the anxiety-like behavior and a total number of open arm and closed-arm entries as an index of overall locomotor activity. Only entering into one arm with all four paws was defined as an entry. The maze was cleaned after each trial to prevent olfactory cues from affecting the behavior.

### Open field test

Open field test was applied before and 4 h after RvD1 treatment to analyze the free locomotor and exploration of rats after different treatments. Animals were placed into one corner of the open field (100 cm × 100 cm × 48 cm). Movement of the animal in the area during the 5-min testing session was recorded. After 5 min, the animal was removed to the home cage, and the open field area was cleaned. Exploration was defined as the time spent in the inner 6 × 6 squares, whereas overall activity was defined as the number of squares crossed during the testing session.

### Pancreatic histology

Rats were deeply anesthetized with sodium pentobarbital (60 mg/kg, *i.p.*) and the pancreas was obtained and then fixed in 4% paraformaldehyde in phosphate buffered (PB, pH 7.4) at 4°C overnight. Pancreatic tissue was then transferred to progressive xylene washes and was placed in cassettes and embedded in paraffin. Paraffin blocks were cut in 5-μm sections and stained with hematoxylin and eosin. Histological sections were analyzed by a pathologist in a double blinded manner. The severity of CP was assessed by semiquantitative scores according to: graded glandular atrophy (0–3); inflammatory cells infiltrations (0–3); intralobular, interlobular and periductal fibrosis (0–3) [25,26].

### Western blot

All animals were rapidly sacrificed and the thoracic (T) 10 spinal cord was rapidly harvested and then was frozen on the dry ice. Then the spinal dorsal horn was quickly micro-dissected. Spinal tissue was then homogenized in SDS sample buffer (10 ml/mg tissue) with a mixture of proteinase and phosphatase inhibitors (Sigma). The electrophoresis samples were heated at 100°C for 5 min and loaded onto 10% SDS-polyacrylamide gels with standard Laemmli solutions (Bio-Rad Laboratories, CA, USA). The proteins were electroblotted onto a polyvinylidene difluoride membrane (PVDF, Immobilon-P, Millipore, Billerica, MA, USA). The membranes were placed in a blocking solution containing Tris-buffered saline with 0.02% Tween-20 (TBS-T) and 3% non-fat milk for 1 h, and incubated overnight under gentle agitation with primary antibodies: rabbit anti-phosphorylated NR1 (pNR1, 1:1000; Millipore, Billerica, MA, USA), rabbit anti-NR1 (1:500; Millipore), rabbit anti-phosphorylated NR2B (pNR2B, 1:500; Millipore), rabbit anti-NR2B (1:500; Millipore), and mouse anti-beta-actin (1:3000; Millipore). Bound primary antibodies were detected with the anti-rabbit or anti-mouse horseradish peroxidase (HRP)-conjugated secondary antibody (1:5,000; Amersham Pharmacia Biotech Inc., Piscataway, NJ, USA). Between each step, the immunoblots were rinsed with TBS-T. All reactions were detected by the enhanced chemiluminescence (ECL) detection method (Amersham). The densities of protein blots were analyzed with Labworks Software (Ultra-Violet Products, UK). The densities of target proteins and beta-actin were quantified with background subtraction. Target protein levels were normalized against beta-actin levels and expressed as relative fold changes compared to the naïve control group.

### Double-labeling immunofluorescent histochemistry

The rats were deeply anesthetized and perfused transcardially with 100 mL of 0.01 M phosphate-buffered saline (PBS, pH 7.4) followed by 500 mL of 4% paraformaldehyde in 0.1 M phosphate buffer (pH 7.4). After the perfusion, the T10 segments were harvested and cryoprotected in 30% sucrose overnight. The transverse sections were cut on a cryostat at a thickness of 30 μm. The sections were incubated overnight with a mixture of rabbit anti-NR1 (1:500, Millipore) with mouse anti-NeuN (1:3000; Millipore), or mouse anti-glial fibrillary acidic protein (GFAP) (1:5000; Millipore), or mouse anti-OX42 (1:500; Millipore); or with a mixture of rabbit anti-NR2B (1:500, Millipore) with mouse anti-NeuN (1:3000; Millipore), or mouse anti-GFAP (1:5000; Millipore), or mouse anti-OX42 (1:500; Millipore). The sections were then washed for 3 times in 0.01 M PBS (10 min each) and then incubated for 2 h at RT with the corresponding secondary antibody: Alexa Fluor 488-conjugated goat anti-rabbit IgG (1:200; Molecular Probes, Eugene, OR, USA) and Alexa Fluor 594-conjugated goat anti-mouse IgG (1:800; Molecular Probes). Images were obtained using a confocal laser microscope (FV1000; Olympus, Tokyo, Japan) and Digital images were captured with Fluoview 1000 (Olympus).

### Real-time RT-PCR

After behavioral tests, rats were rapidly sacrificed and the T10 spinal cord was rapidly harvested and then the spinal dorsal horn was quickly micro-dissected on the dry ice. RNA was extracted with Trizol (GIBCO/BRL Life Technologies Inc., Grand Island, NY, USA). Complementary DNA (cDNA) was synthesized with oligo (dT)_12-18_ using Superscript ™ III Reverse Transcriptase for RT-PCR (Invitrogen, Carlsbad, CA, USA). The primers used were shown in Table 1. One microgram of RNA was used to prepare cDNA using the SYBR® Premix Ex Taq™ (Takara, Tokyo, Japan). Real-time PCR was then performed in a detection system (Applied Biosystems, Foster City, CA, USA). The amplification protocol was: 3 min at 95°C, followed by 40 cycles of 10 s at 95°C for denaturation and 45 s at 60°C for annealing and extension. All experiments were repeated for 3 times and in each experiment, PCR reactions were done in triplicate. Target cDNA quantities were estimated from the threshold amplification cycle number (C_t_) using Sequence Detection System software (Applied Biosystems). GAPDH was served as an endogenous internal standard control for variations in RT-PCR efficiency.

**Table 1:** Primers sequence for the rat genes characterized in this experiment

**Genes**	**Primers**		**Accession number**
TNF-alpha	Forward primer	5’-TGATCGGTCCCAACAAGG A-3’	AY427675
	Reverse primer	5’-TGCTTG GTG GTTTGCTACGA-3’	
IL-1beta	Forward primer	5’-TGCTGATGTACCAGTTGGGG-3’	NM031512
	Reverse primer	5’-CTCCATGAGCTTTGTACAAG-3’	
IL-6	Forward primer	5’-GCCCTTCAGGAACAGCTATG-3’	NM012589
	Reverse primer	5’-CAGAATTGCCATTGCACAAC-3	
GAPDH	Forward primer	5’-CCCCCAATGTATCCGTTGTG-3’	NM01008
	Reverse primer	5’-TAGCCCAGGATGCCCTTTAGT-3’	

#### ELISA

In order to determine protein expression of TNF-alpha, IL-1beta and IL-6, rats were rapidly sacrificed and the T10 spinal cord was rapidly harvested and then the spinal dorsal horn was quickly micro-dissected and homogenized in lysis buffer containing protease inhibitors, and the insoluble pellet was separated out by centrifugation. TNF-alpha, IL-1beta and IL-6 concentration in the supernatants were measured spectrophotometrically using a commercially available ELISA kit according to the manufacturer’s instructions (BioSite, Paris, France). TNF-alpha, IL-1beta and IL-6 were measured at 450 nm using a Fluoroskan Ascent cytofluorimeter (Thermo Electron, Milford, MA, USA), and the concentrations were calculated on the basis of relative recombinant standard curve. All samples were processed in triplicate. Data were obtained from 3 independent experiments.

### Data analysis

All data were collected by experimenters blind to the surgery and treatments and statistical analysis was done by using SPSS software (version 12.0). Data were expressed as means ± standard error of the mean (means ± S.E.M.). Repeated measures ANOVA (with Bonferroni confidence interval adjustment) were used and conducted for data of pancreatic pathology, Western blot and real-time PCR analysis. Dunnett’s C *post hoc* test, assuming the variances were not equal, was employed whenever appropriate and significance was set at 0.05 for all statistical tests.

## Competing interests

The authors declare that they have no competing interests.

## Authors’ contributions

FQ-X and FF contributed equally to this work. FQ-X performed histology and Western blot. FF performed the behavioral testing, immunostaining and statistical analysis. FX-Y performed real-time RT-PCR and ELISA. LS-J helped to perform histology. LZ-X helped to perform behavioral testing. ZX-J helped to draft the manuscript. ZQ-C participated in the experimental design and coordination. WW conceived of the study, and drafted the manuscript. All authors read and approved the final manuscript.

## Pre-publication history

The pre-publication history for this paper can be accessed here:

http://www.biomedcentral.com/1471-230X/12/148/prepub
